# Increased Risk of Asthma and Allergic Rhinitis in Patients With a Past History of Kawasaki Disease: A Systematic Review and Meta-Analyses

**DOI:** 10.3389/fped.2021.746856

**Published:** 2021-12-20

**Authors:** Wei-Te Lei, Chih-Wei Hsu, Po-Cheng Chen, Ping-Tao Tseng, Ho-Chang Kuo, Mindy Ming-Huey Guo, Yu-Kang Tu, Pao-Yen Lin, Yu-Hsuan Kao, Ling-Sai Chang

**Affiliations:** ^1^Department of Pediatrics, Hsinchu MacKay Memorial Hospital, Hsinchu, Taiwan; ^2^Graduate Institute of Clinical Medical Sciences, College of Medicine, Chang Gung University, Taoyuan City, Taiwan; ^3^Department of Psychiatry, Kaohsiung Chang Gung Memorial Hospital and Chang Gung University College of Medicine, Kaohsiung City, Taiwan; ^4^Department of Physical Medicine and Rehabilitation, Kaohsiung Chang Gung Memorial Hospital and Chang Gung University College of Medicine, Kaohsiung City, Taiwan; ^5^Department of Public Health, College of Medicine, National Cheng Kung University, Tainan, Taiwan; ^6^Prospect Clinic for Otorhinolaryngology and Neurology, Kaohsiung City, Taiwan; ^7^Department of Pediatrics and Kawasaki Disease Center, Kaohsiung Chang Gung Memorial Hospital, Kaohsiung City, Taiwan; ^8^College of Medicine, Chang Gung University, Taoyuan City, Taiwan; ^9^Institute of Epidemiology and Preventive Medicine, College of Public Health, National Taiwan University, Taipei City, Taiwan; ^10^Department of Dentistry, National Taiwan University Hospital, Taipei City, Taiwan; ^11^Institute for Translational Research in Biomedical Sciences, Kaohsiung Chang Gung Memorial Hospital, Kaohsiung City, Taiwan; ^12^Section of Immunology, Rheumatology, and Allergy, Department of Pediatrics, Mackay Memorial Hospital, Taipei City, Taiwan

**Keywords:** allergic disease, allergic rhinitis, asthma, atopic dermatitis, Kawasaki disease, meta-analysis

## Abstract

**Background:** Allergic diseases are frequently observed in children with Kawasaki disease (KD). However, the evidence supporting the association between KD and allergies has been conflicting. The objective of the current study is to examine the association between KD and allergic diseases.

**Methods:** We conducted an electronic search using PubMed, Embase, and the Cochrane through 24 July 2021. The inclusion criteria consisted of studies that examined the prevalence of allergic diseases in children with a previous diagnosis of KD and in a comparison group. We pooled studies by using a random effects model. The effects of KD on the subsequent risk of allergic diseases were expressed as odds ratio (OR) with 95% confidence intervals (CI).

**Results:** We included a total of four studies that assessed the effect of KD on asthma, allergic rhinitis, and atopic dermatitis vs. non-KD children (KD individuals for asthma, four studies, *n* = 8,474; allergic rhinitis, four studies, *n* = 8,474; atopic dermatitis, three studies, *n* = *8,330*). The overall prevalence of asthma, allergic rhinitis, and atopic dermatitis was 9.12, 27.63, and 6.55% among patients with previous KD. The meta-analysis showed a significantly increased risk of asthma (OR:1.437, CI: 1.067–1.937) and allergic rhinitis (OR: 1.726, CI: 1.291–2.307) in patients with KD, compared with the control groups. However, patients with KD did not have a significantly different level of risk of atopic dermatitis (OR: 1.243, 95% CI: 0.857–1.802).

**Conclusion:** This meta-analysis supports that individuals with KD are more likely to have asthma and allergic rhinitis compared to controls.

## Introduction

Kawasaki disease (KD) is a form of acute febrile medium vessel vasculitis and the most common cause of acquired heart disease in susceptible children. The cause of KD with a cluster of phenotypes remains unknown, but some evidence supports that KD comes from an exaggerated immune response similar to asthma with type 2 inflammation (1, 2). Studies investigating the involvement of type 2 inflammation in KD revealed eosinophilia and elevation of such mediators as immunoglobulin (Ig)E, interleukin (IL)-4, IL-5, eotaxin, and eosinophil cationic protein (3, 4).

Triggers for KD, such as pollen- or mite-induced delayed-type hyper-sensitivity, may play a role in the pathogenesis of KD (5, 6). Supporting evidence has shown an increased KD risk associated with previous allergic diseases such as asthma, allergic rhinitis, atopic dermatitis, and urticaria in children (7, 8). A previous report found a positive correlation between Japanese cedar pollen numbers and KD development (9). In contrast, another study demonstrated less exposure to allergens before KD, but more exposure to chemical substances (10). Some people think that the increase in IgE and IL-4 is only temporary, so it will not cause an increase in allergic diseases (11).

Recent epidemiological studies have suggested that the prevalence of allergy was elevated in children before or after KD. Eosinophilic asthma before KD was found to be associated with the risk of KD in a retrospective population-based case-control study (7). The prevalence of bronchial asthma at onset and follow-up of KD was higher than the general children's population (12). Upon flare-ups, the asthma improved without hospitalization (11). Other studies by Wei et al. also found a positive association between allergic rhinitis, atopic dermatitis, and the risk of KD (8). One study from Taiwan demonstrated that KD was associated with any concomitant atopic diseases (13). One cross-section study revealed children with a past history of documented KD were at a higher risk for developing nasal problems, itchy eyes, diagnosed rhinitis, wheezing, and nocturnal cough than the general population (14).

However, whether long-term care will show an association between allergic diseases and KD remains unclear. Many physicians mistakenly believe that most patients with KD are recovering well and ignore the importance of following up (15). In fact, the proportion of allergies after KD may be very high, especially allergic rhinitis, which may be as high as 47.04% within 6 years of the KD diagnosis (16). Currently, no research has performed comprehensive statistics of meta-analysis on this topic. We conducted the first meta-analysis, with the aim of investigating the long-term risks of KD patients with allergic diseases.

## Methods

### Literature Search and Screening Strategy

We performed an electronic systematic literature search on the platforms of PubMed, Embase, and the Cochrane library for studies on KD, from inception to 24 July 2021. In the current study, three well-trained authors (L.S. Chang, P.C. Chen, and C.W. Hsu) independently performed a systematic literature search from the study's inception and scanned the outcomes of the search based on the abstracts and titles using a predetermined list of variables of interest.

**Figure 1:**
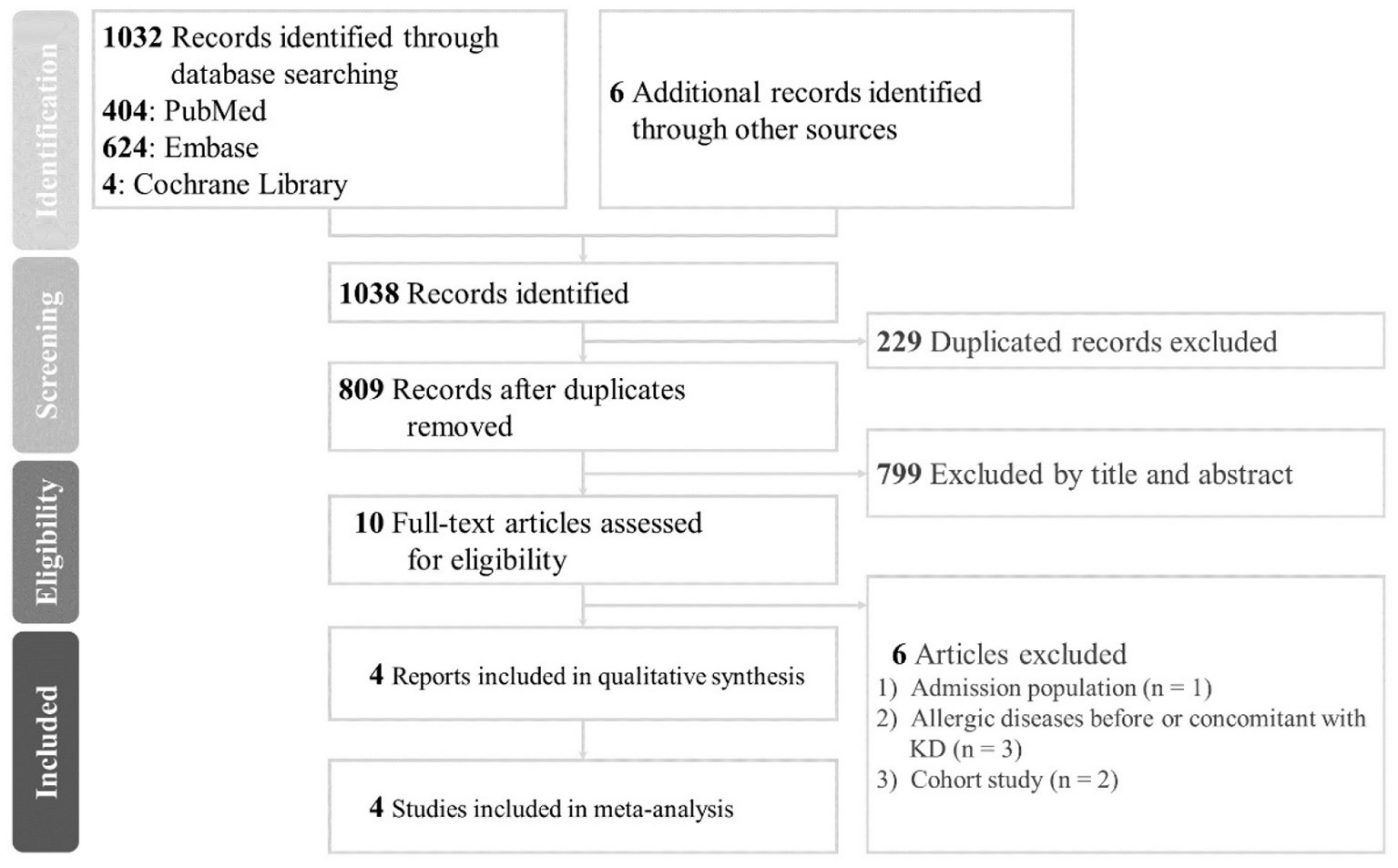
Preferred reporting items for systematic reviews and meta-analysis (PRISMA) 2009 flow diagram; KD, Kawasaki disease.

The search keywords were “Kawasaki disease^*^” AND (allerg^*^ OR atop^*^ OR asthma OR rhinitis OR dermatitis OR eczema) and were adjusted according to each database's requirements (17). The review questions were formulated following the PICO criteria (Population, Intervention, Comparator, and Outcome) in the Embase database using the keywords: “mucocutaneous lymph node syndrome” /exp AND (“allergy” /exp OR “allergic condition” OR “allergic state” OR “allergy” OR “allergy, physical” OR “physical allergy”). The present study was conducted in accordance with the Preferred Reporting Items for Systematic Reviews and Meta-Analysis (PRISMA) (Figure 1; Supplemental Table 1) (18). Furthermore, manual searches were performed for potential papers selected from the reference lists of the included articles to expand our eligible list.

### Eligibility Criteria and Data Extraction

We included both case-control and cross-sectional designs that were conducted in patients after KD diagnosis. Regarding the outcome and exposure measures, any assessment method of KD, asthma, allergic rhinitis, and/or atopic dermatitis was included in the narrative review, as well as in the quantitative meta-analysis. We imposed no date or types of publication restrictions on the search. The exclusion criteria were studies that reported allergic diseases before or concomitant with KD development, studies without distinguishable comparison groups, or articles that did not provide original data or full-texts. We also excluded studies not written in English. An overlapping or similar dataset used in different published articles or a dataset using a cohort study was excluded.

During the screening stage, the three authors screened all of the titles and abstracts of the identified results. Studies not relevant or available to the prevalence or incident rate of allergic diseases including asthma, allergic rhinitis, or atopic dermatitis in patients after KD were excluded in this stage. We subsequently performed examination of full-texts after developing a list of potentially eligible studies.

Between-reviewer inconsistencies concerning eligibility, inclusion, or data extraction were settled through discussion after reading the full text under the supervision of the corresponding authors followed by mutual consensus. We were thus able to determine a final list of included studies.

Either crude data on exposure and outcome or the odds ratio (OR) of the association between an allergic disease and KD needed to be recorded. The independently extracted data of interest comprised information on the study design and characteristics, including the first author, published year, number of participants, sex ratio in the form of the percentage of males, mean age, crude unadjusted study outcome (two by two contingency table), study outcome adjusted for confounding factors (adjusted OR), information on confounders, and the assessment method of both the allergic diseases and KD.

### Primary Outcomes

We set the primary outcomes as the OR of the association between asthma, allergic rhinitis, or atopic dermatitis following KD. We aimed to investigate studies that examined the prevalence and/or incidence of one or more of the three allergic diseases, including asthma, allergic rhinitis, and atopic dermatitis, in subjects with KD compared with those without KD. Data was calculated from the information available in the manuscripts without contacting the original authors.

### Data Synthesis

We investigated the association of KD with the development of the three allergic diseases using Comprehensive Meta-Analysis software 3.3 (CMA, Borenstein, M., Hedges, L., Higgins, J., & Rothstein, H. Biostat, Englewood, NJ 2013). We adopted CMA to determine the potential influences of induvial studies by sensitivity analysis of one-study removement. Subgroup analyses were conducted to determine the difference between research methods. Based on the assumed heterogeneous background and populations among the recruited studies, crude data were pooled using the random effect model meta-analyses to determine the OR with the 95% confidence interval (CI).

### Heterogeneity and Publication Bias

*I*^2^ tests were assessed for statistical heterogeneity in the included studies. Potential publication bias was simultaneously evaluated by visual inspection of funnel plots and Egger's regression test (19).

### Assessment of Study Quality

We used the modified version of the Newcastle-Ottawa Scale (NOS; Stang, 2010) designed to assess the quality of observational studies, ranging from zero (low quality) to ten (high quality) for case-control and cross-sectional studies to determine the methodological quality of the included studies (19–21). No threshold of NOS has been validated. According to a previous meta-analysis, we defined studies as being low quality (score <5), moderate quality (score 5–6), or high quality (score >6) (22).

## Results

### Identification and Selection of Studies

In this review, the total yield was 1,038 articles, of which 229 duplicate studies were removed. We then excluded 799 studies based on their titles and abstracts, leaving 10 records to be retrieved for further screening. Only four studies were not based on the Taiwan Health Insurance Database, two of which were cross-sectional studies, and the other two were case-control studies (23–26). The majority of cross-sectional and longitudinal studies reported a statistically significant positive association. On reviewing full-text articles, we excluded two cohort studies duplicated with the research of the Taiwan Health Insurance Database, one that included only admission population, and three studies that included participants with allergic diseases before or concomitant with KD onset (4, 7, 8, 13, 16, 26). Four studies in four countries representing 8,474 participants were finally included in the meta-analysis (23–25, 27). The proposed algorithms with the number of studies are illustrated in Figure 1.

The main characteristics and population of the four studies included in the meta-analysis are depicted in Table 1. Regarding types of allergic diseases assessed among patients with KD, one study included only the outcome of asthma and allergic rhinitis (23). CMA was performed for the results of asthma, allergic rhinitis, and atopic dermatitis after KD. The studies included in this systematic review were published between *1997* and 2016, and the sample size of KD patients ranged from *9* 3 in Singapore to 7,072 in Taiwan, including *5,304* males and *3,170* females. More than half of the participants were males (62.59% in KD children and 58.58% in controls). The average number and standard error of subjects was *2,118*.50 ± *1,669*.52. Of the four studies included in this review, one was from Israel, one from Singapore, one from Japan, and one from Taiwan, with varied data sources. In this analysis, the vast majority were Asians, including Chinese, Indians, Japanese, and Malays (24, 25, 27). Studies used such assessment methods as interview via telephone, questionnaire (parental report or ISAAC, i.e., the International Study of Asthma and Allergies in Childhood), code in database, or medical records. The number of the studies and sample size for each allergic disease were as follows: asthma, four studies, *n* = 8,474; allergic rhinitis, four studies, *n* = 8,474; atopic dermatitis, three studies, *n* = *8,330*.

**Table 1:** Included studies looking into the Kawasaki disease and subsequent different allergic diseases.

**References**	**Study design**	**Location**	**Outcome (95% CI)**	***N* of KD**	**Age at diagnosis (years)**	**Age at study (years)**	**Gender (male%)**	**Types of allergic diseases**	**Outcome measurements**	**Comparator**	**The number of control subjects**	**The methods of diagnosing KD**	**NOS**
Matsuoka et al. (25)	CASE-CONTROL	Japan	Relative risk	1,165	Between 1 and 5	6–18	64.46	Asthma AR AD	Parental questionnaire	Non KD	5,825	The Japanese Kawasaki Disease Research committee	6
Liew et al. (24)	CROSS-SECTION	Singapore	Adjusted OR 2.56 (0.80–8.23) 2.90 (1.27–6.60) 1.30 (0.52–3.23)	93	1.17	7.08	58	Asthma AR AD	ISAAC questionnaire	Well sibling	93	Not reported	9
Tsai et al. (27)	CASE-CONTROL	Taiwan	Adjusted OR 1.03 (0.93–1.13) 1.28 (1.20–1.37) 0.95 (0.82–1.10)	7,072		6–13	62.09	Asthma AR AD	Code in database	Non KD	27,265	ICD-9-CM code: 446.1	6
Hassidim et al. (23)	CROSS-SECTION	Israel	OR 2.92 (1.68–5.07) 2.63 (1.69–4.11)	144		17	75	Asthma AR	Code in medical records	General population	1,187,757	FFS classification Numerical codes	7

*AD, atopic dermatitis; AR, allergic rhinitis; CI, confidence interval; FFS, fitness-for service; ICD, International Classification of Diseases; ISAAC, the International Study of Asthma and Allergies in Childhood; KD, Kawasaki disease; N, number; NOS, Newcastle-Ottawa Scales; OD, odds ratio*.

### Association Between Kawasaki Disease and Asthma

**Figure 2:**
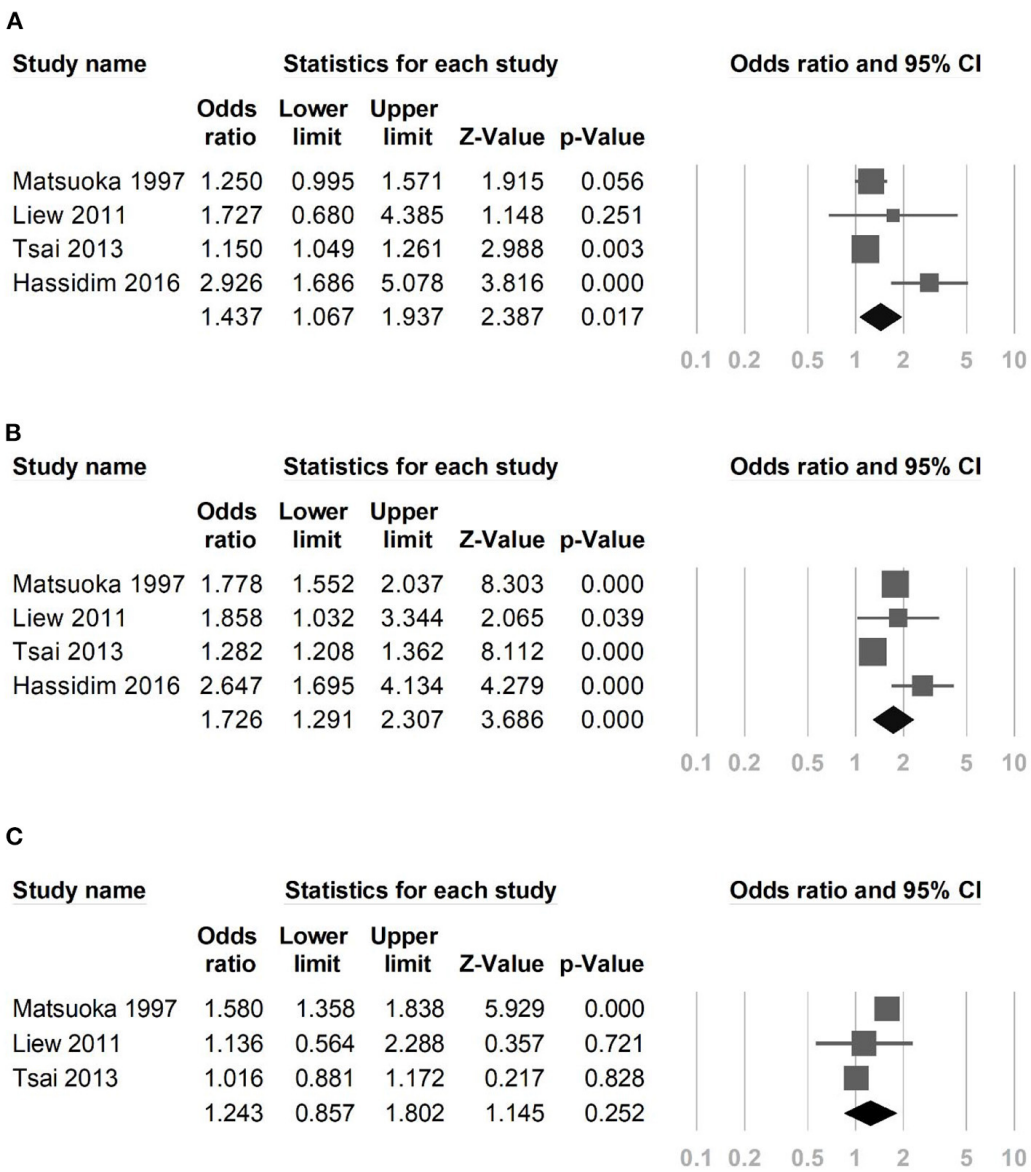
Forest plot in the meta-analysis of **(A)** asthma among patients with Kawasaki disease, **(B)** allergic rhinitis among patients with Kawasaki disease, **(C)** atopic dermatitis among patients with Kawasaki disease.

Four studies that reported asthma among patients with KD were included in the final analysis (Table 1). Of the 8,474 children with Kawasaki disease, 773 were diagnosed with asthma. Based on the results of the random-effects meta-analysis model, the pooled estimated OR of children having KD compared to those without was 1.437 (95% CI 1.067–1.937), demonstrating a statistically significant difference (*p* = 0.017) and indicating an association between KD and asthma. We found medium heterogeneity for this analysis (*I*^2^ = 74%; *p* = 0.009) (see Figure 2A). To explore the influence of case-control studies vs. cross-sectional studies on the effect estimate, we conducted subgroup analysis. The result from the subgroup analysis showed that a higher proportion of children in the cross-sectional design subgroup (two studies, OR: 2.552, 95% CI: 1.588–4.102) had asthma compared to the children in the case-control subgroup (two studies, OR: 1.164, 95% CI: 1.069–1.267, *p* = 0.001).

### Association Between Kawasaki Disease and Allergic Rhinitis

Four studies examined the risk of allergic rhinitis in children with KD compared to children without KD. The meta-analysis showed that children with KD were likely to have allergic rhinitis compared with those in the control groups (OR: 1.726; 95% CI; 1.291–2.307). We found an apparent heterogeneity among the studies included in this meta-analysis (*I*^2^ = 89%; *p* < 0.001) (see Figure 2B). Regarding subgroup analysis, no apparent difference was detected between the case-control and cross-sectional studies (*p* = 0.070).

### Association Between Kawasaki Disease and Atopic Dermatitis

The summary OR of atopic dermatitis among KD patients was 1.243 (95% CI: 0.857–1.802; three studies, *n* = 8,330 KD children) (Figure 2C). This finding showed that, while children with KD have more atopic dermatitis compared with those in the control groups, it does not reach statistical significance. The heterogeneity across the studies was found to be statistically significant (*I*^2^ = 89%; *p* < 0.001).

### Sensitivity Analysis

In the meta-analysis of the asthma group, removing studies performed by Matsuoka et al. or Tsai et al. affected the asthma risk following KD (Supplemental Figure 1A) (25, 27). Both research groups contain more than 1,000 KD children. Removing any study did not affect the significance in risk of allergic rhinitis following KD (Supplemental Figure 1B). In other words, the significance was not impacted by only one study. In the meta-analysis of the effect of KD on the subsequent risk of atopic dermatitis, removing the study by Tsai et al. increased the risk of atopic dermatitis following KD (Supplemental Figure 1C) (27). Considering that the ages of atopic dermatitis and KD are similar or even younger, if we replace the research in the Taiwan Health Insurance Database with atopic dermatitis comorbid with KD, the main results of meta-analysis change into significantly higher co-morbidity rates of AD in subjects with KD from OR: 1.243, 95% CI: 0.857–1.802 to OR: 1.555, 95% CI: 1.175–2.057 (13). The meta-analysis showed that children experienced 55% greater odds of having AD before, after, or along with KD compared with those without KD.

### Methodological Quality Appraisal of Included Studies and Publication Bias

Across the four studies, the average NOS for quality assessment was 7, as shown in Supplemental Table 2. Of the included studies, two studies were of high quality with NOS score 7 and 9, and two were of moderate quality with NOS score 6. Egger's tests did not show any risk of publication bias (*p* = 0.183 for asthma, *p* = 0.203 for allergic rhinitis, *p* = 0.991 for atopic dermatitis) in Supplemental Figure 2.

## Discussion

An association between previous KD and asthma and allergic rhinitis was identified in the current meta-analysis, but other potential risk factors like family history or environment were not fully illustrated or corrected. The enrolled studies observed an association between KD and allergic diseases up to 18 years old. In contrast, no association between atopic dermatitis and KD was found. The possible reason is that children with atopic dermatitis are younger, even younger than KD patients. Our analyses based on a case-control study of concurrent atopic dermatitis suggest increased risks of atopic dermatitis in relation to KD. Similarly, longitudinal analysis of Taiwanese cohorts followed for 6 years found consistent evidence that KD was associated with a higher risk of asthma and allergic rhinitis (16). However, in the analyses based on atopic dermatitis risk after KD, a cohort study performed by Woon et al. found higher risks of atopic dermatitis in relation to KD at the 5-year follow-up (4). The possible reason is that the tracking time of the cohort study was short, and older children were not considered.

Dysbiosis related Treg (regulatory)/T helper type (Th)17 imbalance and type 2 inflammation may explain part of the immunopathogenic connection between KD and allergic diseases (28, 29). However, there are notable differences, with the role of infection supported by global investigation of immune repertoire and inflammatory responses especially cytokines storms in the pathogenesis of KD (30, 31). Given the roles of IgA antibodies in mucosal immunity and Fc receptor for IgA encoded by *FCAR* in triggering IgA-mediated immune responses to pathogens, increasing evidence has shown that a FcαR-mediated immune system is related to inflammatory and infectious diseases (32). The gastrointestinal permeability increased in KD, and the deposition of IgA in the blood and vascular tissues also increased (33). Eosinophils are required to generate and maintain mucosal IgA plasma cells for mucosal immune homeostasis (34, 35). In eosinophil-deficient mice, the production of IgA was reduced (36). Eosinophils from small intestinal were demonstrated to regulate adaptive humoral IgA responses (37). The number of cells producing IgA increased in KD (38). After intravenous immunoglobulin (IVIG) treatment, IgA increased remarkably (39). The transcriptome array showed that the transcriptional levels of the IgA receptor, *FCAR* was significantly higher in KD and decreased after IVIG (2). The final product of lung-resident eosinophils is L-selectin which tends to be higher during the convalescent phase of KD than in either the acute or the subacute phase (40). Plasma L-selectin levels in patients with coronary artery lesions (CAL) tend to be lower than those in patients without CAL. The finding is consistent with lower eosinophils in patients with CAL. Eosinophilic chronic rhinosinusitis (ECRS) is one kind of rhinitis characterized by prominent eosinophilic infiltration. L-selectin plays a role in eosinophil recruitment in human nasal mucosa of ECRS (41). Aberrant production of inflammatory cytokines including tumor necrosis factor α and IL-6 promoted leukocyte-endothelial cell interactions that caused endothelial dysfunction then leading to the development of allergic diseases in KD (42, 43).

Liew et al. identified KD children without CAL have an intensified form of allergic rhinitis and any other allergies when compared with controls, consistent with lower Th2 cytokines in KD patients with CAL (3, 24). No research reports were available regarding the relevance of drugs (IVIG, corticosteroids, aspirin, etc.) used to treat KD on allergic diseases. In mice, IVIG caused an increase in inhibitory receptors through type 2 inflammatory cytokine IL-4 (44, 45). Whether in enterovirus or KD, eosinophil counts were found to be elevated following IVIG administration (46). However, some patients included in the study among our meta-analysis may have been collected earlier than the evidence-recommended IVIG therapy (47). Corticosteroids also treat type 2 inflammation, so it is curious whether the drug will affect allergic diseases in patients with a past history of KD (48, 49). Since these studies have not shown the use of corticosteroids in patients, we cannot analyze the effects of corticosteroids on allergic diseases (48). High- or low-dose aspirin is often used in the treatment of KD (50). Very few studies have addressed aspirin-induced respiratory diseases that affect people between the ages of 20 and 50 and cause breathing problems such as asthma and sinus problems with nasal polyps (51, 52). The research of the Taiwan National Health Insurance Database cannot prove that the prevalence of asthma in children was higher in children using aspirin (53). The cohort study demonstrated that the use of aspirin or non-steroidal anti-inflammatory drugs increased admission for asthma exacerbation. Therefore, although we have observed that the risk of asthma and allergic rhinitis increases after KD, we cannot determine whether it is due to aspirin treatment or not. Further larger scale prospective surveys are needed to answer this important issue.

This study has several limitations. First, taking into account the duplicate database researches and the comparability of data, this meta-analysis included only four studies with case-control/cross-section designs, and no cohort studies were enrolled. Therefore, we were unable to further investigate the relationship between the gender, ethnicity, and age differences at the time of KD diagnosis and the subsequent development of allergic diseases or the increasing risk per year following KD for each disease (8, 25). Second, KD and outcomes of allergic diseases lack a rigorous definition in this meta-analysis (54, 55). For example, the four included studies have different methods for determining KD (Table 1), and we cannot rule out the cases with incomplete KD through the diagnosis code. The absence of homogeneity in the definition of KD may hinder clinical interpretation.

In conclusion, the results of this meta-analysis provided consistent evidence of an association between KD and subsequent asthma and allergic rhinitis. Physicians should be aware of the distinct clinical presentations of allergic diseases and set up a tracking plan of KD even to adulthood.

## Data Availability Statement

The raw data supporting the conclusions of this article will be made available by the authors, without undue reservation.

## Author Contributions

Three well-trained authors (L-SC, P-CC, and C-WH) independently performed a systematic literature search from the study's inception and scanned the outcomes of the search. During the screening stage, the three authors (W-TL, Y-HK, and P-TT) screened all of the titles and abstracts of the identified results. L-SC, H-CK, and MG: conceptualization. P-CC and C-WH: methodology. Y-KT and P-YL: validation. L-SC and C-WH: formal analysis. L-SC: writing—original draft preparation. P-YL and C-WH: writing—review and editing. Y-KT and P-YL: supervision. L-SC: funding acquisition. All authors have read and agreed to the published version of the manuscript and drafted the work or substantively revised it, have approved the submitted version.

## Funding

This study was supported in part by Chang Gung Memorial Hospital (CFRPG8K0051,61,71,81, CMRPG8K0642) and the Ministry of Science and Technology, Taiwan (NMRPG8L0061). However, these institutions had no role in the study design, data collection and analysis, decision to publish, or preparation of the manuscript.

## Conflict of Interest

The authors declare that the research was conducted in the absence of any commercial or financial relationships that could be construed as a potential conflict of interest.

## Publisher's Note

All claims expressed in this article are solely those of the authors and do not necessarily represent those of their affiliated organizations, or those of the publisher, the editors and the reviewers. Any product that may be evaluated in this article, or claim that may be made by its manufacturer, is not guaranteed or endorsed by the publisher.
